# Optimization of Spatial Downscaling Models for Satellite Imagery Based on Deep Learning and Generative Artificial Intelligence

**DOI:** 10.3390/s26196147

**Published:** 2026-09-28

**Authors:** Juan Valdés-Quintero, Rubén Darío Vásquez-Salazar, Juan Camilo Parra, César Olmos-Severiche, Andrés Gustavo Camargo-Perea, Cristian Alejandro Tibavija-Abril, Jean Pierre Díaz-Paz

**Affiliations:** 1 Faculty of Engineering, Politécnico Colombiano Jaime Isaza Cadavid, 48th Av, 7-151, Medellín 050022, Colombia; jcparra@elpoli.edu.co (J.C.P.); cesarolmos@elpoli.edu.co (C.O.-S.); andres_camargo76222@elpoli.edu.co (A.G.C.-P.); cristian_tibavija76231@elpoli.edu.co (C.A.T.-A.); 2 Faculty of Engineering, Universidad Militar Nueva Granada, km2 Cajicá—Zipaquirá, Cajicá 250247, Colombia; jean.diaz@unimilitar.edu.co

**Keywords:** remote sensing, downscaling, deep learning, computer vision, super resolution

## Abstract

**Highlights:**

**What are the main findings?**

- Off-the-shelf pretrained super-resolution models (ESRGAN, SwinIR) applied directly to Landsat-to-Sentinel-2 upscaling do not outperform bicubic interpolation on pixel-wise metrics, remaining statistically indistinguishable from it (Nemenyi post hoc), thereby revealing a domain adaptation gap.
- Domain-specific fine-tuning with only 3000 paired Landsat–Sentinel-2 patches completely reverses this degradation: ESRGAN (train 2) achieves the best performance across all five evaluation metrics simultaneously (MSE, PSNR, SSIM, LPIPS, DISTS), with a 48.7% MSE reduction and a 1.84 dB PSNR gain over the pretrained baseline.

**What are the implication of the main findings?**

- Practitioners applying natural-image super-resolution models to satellite data without domain-specific fine-tuning risk systematically degrading radiometric fidelity, which is critical for applications such as spectral change detection, vegetation index computation, and radiometric calibration.
- A hybrid evaluation framework combining pixel-wise metrics (MSE, PSNR, SSIM) with perceptual metrics (LPIPS, DISTS) and non-parametric statistical validation (Friedman–Nemenyi) is necessary for reliable model comparison in satellite super-resolution, as these metric families can diverge and no single metric suffices to characterise reconstruction quality.

**Abstract:**

Spatial downscaling of satellite imagery, the reconstruction of high-resolution outputs from coarser-resolution inputs, is a critical enabler of long-term land cover monitoring, yet the domain adaptation gap between natural-image super-resolution models and satellite sensor characteristics remains largely unaddressed. This study proposes a transfer learning framework for Landsat-to-Sentinel-2 spatial downscaling at a scale factor of ×3, systematically comparing shallow and deep fine-tuning strategies applied to two state-of-the-art architectures: SwinIR and ESRGAN. All models were trained and evaluated on a newly constructed dataset of 3500 paired patches assembled through an automated Google Earth Engine pipeline with rigorous cloud, water, and spectral variability filters spanning latitudes −30° to 30°. Performance is assessed using a hybrid evaluation framework combining pixel-wise metrics (MSE, PSNR, SSIM) with perceptual metrics (LPIPS, DISTS), and statistical significance is established through Friedman tests with Nemenyi post hoc analysis. Results demonstrate that off-the-shelf pretrained models fail to outperform bicubic interpolation on pixel-wise metrics, remaining statistically indistinguishable from it, thereby revealing a domain adaptation gap. Domain-specific fine-tuning completely reverses this degradation: ESRGAN with deep fine-tuning achieves the best performance across all five metrics simultaneously, reducing MSE by 48.7% and improving PSNR by 1.84 dB relative to the pretrained baseline, with all improvements statistically confirmed at p<0.0001. The findings establish that intermediate unfreezing of feature extraction blocks represents the optimal adaptation strategy, and that pixel-wise and perceptual metric families can diverge, making hybrid evaluation a methodological necessity rather than a redundancy.

## 1. Introduction

Land cover monitoring constitutes a cornerstone for understanding environmental transformations, including urban sprawl, deforestation, and the dynamics of water bodies [1,2,3,4,5,6,7]. Remotely sensed imagery has emerged as the primary data source for such analyses, thanks largely to open-access missions such as Landsat and Sentinel, from the American (NASA) and European (ESA) space agencies, respectively [8,9,10]. These programs have generated an unprecedented multi-decadal archive that supports change detection across diverse spatial and temporal scales [4,5,10].

Nevertheless, a fundamental structural limitation persists: no single satellite mission simultaneously offers both high spatial resolution and a long historical record. Sentinel-2 delivers fine 10 m pixels, but its coverage started only in 2015 [9]. In contrast, Landsat provides a continuous record dating back to the 1970s, yet at a three-times coarser resolution of 30 m, available since the launch of Landsat 4 in 1982 and maintained through all subsequent missions to the present day [8,10]. This gap forces analysts to choose between spatial detail (using only recent Sentinel-2 images) or long historical record (using only Landsat), thereby hindering precise assessments of long-term land transformations.

Given these differences in spatial and temporal resolution, large-scale studies with high scientific rigor have generally relied on the Landsat program as their primary data source because of its unparalleled historical archive [10,11]. Notable examples include the Global Surface Water dataset developed by Pekel et al. [4], which monitors long-term changes in the Earth’s surface water, and the global forest cover and forest change assessment by Hansen et al. [5], which quantifies forest cover dynamics and deforestation trends at the planetary scale. While the long temporal record of Landsat makes such analyses possible, its 30 m spatial resolution limits the detection of fine-scale changes that are readily observable with Sentinel-2 imagery. Consequently, many landscape dynamics occurring below the Landsat spatial resolution remain undetected, including variations in small water bodies, narrow riparian features, and localized forest disturbances such as those associated with small-scale agricultural expansion, crop rotation, or selective logging. These sub-pixel processes may represent a substantial amount of environmental information that is lost when analyses rely exclusively on Landsat imagery.

To overcome this trade-off, spatial downscaling, also known as super-resolution, has gained traction. Early approaches relied on statistical interpolation methods (e.g., bicubic, bilinear), which often produce over-smoothed outputs with blurred edges and loss of texture [12,13]. Within remote sensing, downscaling has been proposed as a means of enhancing the utility of Earth observation data when the native spatial resolution is insufficient to characterize environmental processes of interest [13]. Recently, deep learning has revolutionised the field. Architectures based on Convolutional Neural Networks (CNNs) [12], Generative Adversarial Networks (GANs) [14,15], and vision transformers [16,17] have demonstrated remarkable ability to reconstruct high-frequency details from low-resolution inputs.

Despite these advances, a fundamental issue remains largely unaddressed: domain adaptation. Most state-of-the-art super-resolution models are pre-trained on natural image datasets (e.g., DIV2K [18,19], ImageNet [20]), whose statistical properties differ significantly from those of satellite images. Satellite sensors exhibit unique radiometric characteristics, including variable spectral response functions, atmospheric scattering, and surface reflectance anisotropy. Consequently, the direct application of a standard model to Landsat-Sentinel image pairs often results in suboptimal performance, which sometimes even degrades pixel-level fidelity metrics, such as PSNR, even though perceptual quality may appear improved.

This paper addresses the domain adaptation gap by proposing a transfer learning framework tailored to satellite super-resolution. Specifically, we adapt the SwinIR transformer architecture [16], originally trained for natural image restoration, to the specific domain of Landsat-to-Sentinel upscaling. We systematically compare two fine-tuning strategies: (i) a shallow adaptation that adjusts only the upsampling layers, and (ii) a deeper adaptation that also unfreezes the last Swin Transformer block. Additionally, we include Real-ESRGAN [15] as a strong GAN-based baseline. All models are trained and evaluated on a newly constructed dataset of 3500 paired patches (low-resolution Landsat, high-resolution Sentinel-2) assembled through an automated pipeline on Google Earth Engine.

The main contributions of this work are:

- A reusable, paired Landsat-Sentinel dataset with global geographic coverage, accompanied by cloud, water, and spectral variability filters.
- A systematic comparison of shallow vs. deep fine-tuning for transformer-based super-resolution in the remote sensing context, quantifying the impact of unfreezing different components of SwinIR.
- Evidence that pre-trained natural-image models fail to improve pixel-wise metrics (PSNR, MSE) over bicubic interpolation when applied off-the-shelf—performing on par with it—highlighting the necessity of domain adaptation – a finding often overlooked in the literature.
- Comprehensive evaluation using both traditional metrics (MSE, PSNR, SSIM) and perceptual metrics (LPIPS [21], DISTS [22]), revealing that these two families do not always evolve in the same direction.
- Statistical validation confirming that deep fine-tuning yields statistically significant improvements over both the pre-trained model and bicubic interpolation (p<0.01).

The remainder of this paper is organized as follows. Section 2 describes the dataset construction, the model architectures, and the evaluation metrics. Section 3 details the experimental setup, including hyper-parameters and training protocols. Section 3 presents quantitative and qualitative results. Section 4 discusses the findings, compares them with the state of the art, and highlights the novelty of our approach. Section 5 concludes with limitations and future work.

## 2. Materials and Methods

### 2.1. The Dataset

A dedicated dataset of low-resolution (LR) and high-resolution (HR) image patches was constructed to enable supervised training of super-resolution models. The LR images were sourced from the Landsat 8/9 mission (Collection 2, Level-2 surface reflectance), which provides a nominal spatial resolution of 30 m and an uninterrupted global archive since the 1970s [8]. The HR counterparts were taken from Sentinel-2 (Level-2A surface reflectance), offering 10 m resolution but available only from 2015 onward [9]. By combining these two sensors, we exploit the long temporal coverage of Landsat and the fine spatial detail of Sentinel-2, with a consistent scale factor of three.

An automated acquisition pipeline was developed in Python 3.13.15 using the Google Earth Engine (GEE) application programming interface (API). The workflow follows five main steps: (i) random generation of a region of interest (ROI) within the latitude band [−30°,30°] to avoid polar areas; (ii) query of the Sentinel-2 and Landsat collections over the ROI; (iii) application of quality filters; (iv) temporal pairing with a maximum allowed difference of five days; and (v) resampling to fixed patch dimensions and storage.

To guarantee the quality and diversity of the dataset, three filtering criteria were enforced:

- Cloud cover: Only scenes with a cloud pixel fraction ≤1% were retained, estimated from the QA60 band (Sentinel-2) and the QA_PIXEL band (Landsat).
- Water cover: The Normalized Difference Water Index (NDWI) [23,24] was computed; scenes where water pixels exceeded 50% of the ROI were discarded.
- Spectral variability: The coefficient of variation (CV) of the RGB bands was required to be ≥20% to avoid homogeneous, low-information landscapes [25].

Once a valid pair was selected, the images were radiometrically calibrated to surface reflectance (already provided in Level-2 products) and then resampled to a common patch size using area-based resampling (average pooling). To construct three-channel RGB color composites suitable for visualization and deep learning architectures designed for three-channel inputs, we selected specific optical spectral bands corresponding to the visible spectrum for both sensors. For Sentinel-2, bands B4 (Red, 665 nm), B3 (Green, 560 nm), and B2 (Blue, 490 nm) were mapped to the R, G, and B color channels, respectively. For Landsat, we used bands B4 (Red, 655 nm), B3 (Green, 561 nm), and B2 (Blue, 482 nm). The raw surface reflectance band matrices were normalized to the range [0,255] across all three channels to form the final RGB image patches, effectively combining multispectral information into a unified spatial representation while retaining perceptual visual characteristics.

The HR patches (Sentinel-2) have dimensions 192×192 pixels, while the LR patches (Landsat) are 64×64 pixels, exactly preserving the scale factor of three. The final dataset consists of 3000 training pairs and 500 independent test pairs.

### 2.2. Models Architectures

To address the spatial downscaling challenge from Landsat (30 m) to Sentinel-2 (10 m) imagery, this study evaluates two state-of-the-art deep learning paradigms: SwinIR and ESRGAN. Both architectures represent modern single-image super-resolution but rely on distinct computational mechanisms—local self-attention and adversarial learning, respectively—making them the ideal candidates to explore trade-offs between pixel-wise fidelity and high-frequency structural recovery.

### 2.3. Swin Transformer for Image Restoration (SwinIR)

This model is selected due to its capability to capture long-range pixel dependencies and structural patterns without suffering from the heavy computational overhead typical of standard Vision Transformers (ViTs) [16]. By replacing global self-attention with a localized shifted window mechanism, SwinIR effectively handles high-resolution image patches while preserving fine-grained radiometry.

The network processes a low-resolution input image $I_{LR}\in R^{H\times W\times C}$, where *H* and *W* denote the spatial height and width of the image, respectively, and *C* represents the number of spectral channels, through three main stages: Shallow Feature Extraction, Deep Feature Extraction, and High-Quality Image Reconstruction.

#### 2.3.1. Shallow Feature Extraction

This stage is a convolution layer that extracts the initial feature maps $F_{0}\in R^{H\times W\times D}$ (Equation (1)):

(1) $$F_{0}=H_{SF}\left(I_{LR}\right),$$

where $H_{SF}\left(\;\cdot \;\right)$ denotes the convolution operation, and *D* is the number of channels in the shallow feature representation.

#### 2.3.2. Deep Feature Extraction

The features $F_{0}$ are processed by the deep feature extraction module $H_{DF}\left(\;\cdot \;\right)$, which consists of a sequence of *K* Residual Swin Transformer Blocks (RSTBs) followed by a convolutional layer (Equation (2)):

(2) $$F_{DF}=H_{DF}\left(F_{0}\right)$$

where $F_{DF}$ denotes the resulting deep feature representation.

Each RSTB consists of multiple Swin Transformer Layers utilizing multi-head self-attention inside localized, non-overlapping windows of size M×M. Given a local window feature $X\in R^{M^{2}\times D}$, the Window Multi-Head Self-Attention (W-MSA) calculates attention matrices using Query (*Q*), Key (*K*), and Value (*V*) projections (Equation (3)):

(3) $$\mathrm{Attention}\left(Q,K,V\right)=\mathrm{SoftMax}\left(\frac{QK^{T}}{\sqrt{d}}+B\right)V,$$

where *d* represents the query/key dimension, and *B* is a learnable relative position bias matrix. To enable communication between independent boundaries across consecutive layers, the grid is shifted by $\left(\left\lfloor \frac{M}{2}\right\rfloor ,\left\lfloor \frac{M}{2}\right\rfloor \right)$ pixels, introducing Shifted Window Multi-Head Self-Attention (SW-MSA).

#### 2.3.3. Image Reconstruction

Finally, the deep features $F_{DF}$ and shallow features $F_{0}$ are aggregated to reconstruct the high-resolution output $I_{HR}$ via sub-pixel convolution upsampling (Equation (4)):

(4) $$I_{HR}=H_{REC}\left(F_{DF}+F_{0}\right)$$

### 2.4. Enhanced Super-Resolution GAN (ESRGAN)

While SwinIR prioritizes deterministic pixel-wise fidelity, GANs are particularly effective at synthesizing high-frequency spatial details and realistic textures (such as urban boundaries, agricultural rows, and complex geomorphology) that are inherently degraded or lost at lower resolutions. In adversarial super-resolution models, generator networks utilize learned spatial priors from training distributions to reconstruct plausible fine-grained structures rather than visually oversmoothed outputs. ESRGAN [26] improves upon conventional GAN-based super-resolution systems by eliminating Batch Normalization (BN) layers, which tend to introduce unwanted artifacts and limit generalization, and incorporating a relativistic discriminator. The framework is mathematically formulated as a min-max optimization game between a Generator (*G*) and a Discriminator (*D*).

#### 2.4.1. Generator

*G* maps the low-resolution Landsat patch to a Sentinel-2 scale. Its backbone relies on Residual-in-Residual Dense Blocks (RRDB), which combine multi-level residual networks and dense connections without BN layers. The mathematical optimization of the generator is driven by a composite loss function (Equation (5)):

(5) $$L_{G}=L_{percep}+\lambda L_{Ra}+\eta L_{1},$$

where $L_{1}=E{∥G\left(I_{LR}\right)-I_{HR}∥}_{1}$ ensures pixel-level content alignment, $L_{percep}$ penalizes deep feature discrepancies extracted via a pre-trained network, and $L_{Ra}$ is the adversarial component. The parameters λ and η act as balancing coefficients.

#### 2.4.2. Discriminator

Unlike standard discriminators that evaluate a single image in isolation, ESRGAN adopts a Relativistic Average Discriminator ($D_{Ra}$). It estimates the probability that a real high-resolution image $I_{HR}$ is relatively more realistic than a generated image $I_{SR}=G\left(I_{LR}\right)$ on average (Equation (6)):

(6) $$D_{Ra}\left(I_{HR},I_{SR}\right)=\sigma \left(C\left(I_{HR}\right)-E_{I_{SR}}\left[C\left(I_{SR}\right)\right]\right),$$

where C(I) represents the non-transformed discriminator output, E[·] denotes the operation of taking the average over a mini-batch, and σ is the sigmoid activation function.

The adversarial loss optimization for the generator ($L_{Ra}^{G}$) and the discriminator ($L_{Ra}^{D}$) are subsequently minimized using Equation (7) and Equation (8) respectively:

(7) $$L_{Ra}^{D}=-E_{I_{HR}}\left[\log\left(D_{Ra}\left(I_{HR},I_{SR}\right)\right)\right]-E_{I_{SR}}\left[\log\left(1-D_{Ra}\left(I_{SR},I_{HR}\right)\right)\right]$$

(8) $$L_{Ra}^{G}=-E_{I_{HR}}\left[\log\left(1-D_{Ra}\left(I_{HR},I_{SR}\right)\right)\right]-E_{I_{SR}}\left[\log\left(D_{Ra}\left(I_{SR},I_{HR}\right)\right)\right]$$

This optimization enables ESRGAN to reconstruct visually sharp, edge-enhanced geographic features, establishing a robust baseline against SwinIR’s conservative, high-fidelity reconstructions.

### 2.5. Metrics

To rigorously assess the performance of the re-trained models in downscaling Landsat data to Sentinel-2 resolutions, a comprehensive suite of quantitative evaluation metrics is employed. Measuring the fidelity of super-resolution satellite imagery requires balancing pixel-wise radiometry with human-perceived structural and textural realism. Therefore, this study adopts a hybrid approach utilizing three classic mathematical metrics (MSE, PSNR, and SSIM) alongside two state-of-the-art deep learning perceptual metrics (LPIPS and DISTS) [22,27].

The spatial co-registration between Landsat and Sentinel-2 imagery relies on the native orthorectification and geometric alignment provided directly by Google Earth Engine during data extraction. Although a residual baseline registration error is inherently present when pairing cross-sensor satellite data, all quantitative comparisons are consistently evaluated against the same Sentinel-2 high-resolution ground-truth reference (*Y*). Consequently, this baseline misalignment affects all evaluated approaches equally. The comparative framework evaluates the performance relative to a standard bicubic interpolation baseline (non-AI rescaled), followed by the deep learning architectures: low-resolution inputs, ground-truth high-resolution images, bicubic interpolation, pretrained ESRGAN, ESRGAN (train 1), ESRGAN (train 2), pretrained SwinIR, SwinIR (train 1), and SwinIR (train 2).

Given the reference ground-truth high-resolution image (Sentinel-2) denoted as *Y* and the model-generated super-resolution image denoted as $\hat{Y}$, both of size H×W with *C* spectral channels, the evaluation metrics are described as follows.

#### 2.5.1. Mean Squared Error (MSE)

The Mean Squared Error is the baseline metric used to calculate the absolute pixel-wise discrepancy between the reconstructed and target geographic scenes. It computes the average squared difference across all pixel coordinates and spectral bands (Equation (9)).

(9) $$\mathrm{MSE}=\frac{1}{HWC}\sum_{c=1}^{C}\sum_{i=1}^{H}\sum_{j=1}^{W}{\left(Y\left(i,j,c\right)-\hat{Y}\left(i,j,c\right)\right)}^{2},$$

where Y(i,j,c) and $\hat{Y}\left(i,j,c\right)$ represent the reference and reconstructed 8-bit digital number pixel intensity values within the range [0,255] at spatial coordinates (i,j) for spectral band *c*. While MSE is crucial for measuring physical radiative alignment, its value is highly sensitive to outlier pixel variances and fails to capture spatial correlation. A lower MSE indicates tighter mathematical proximity to the reference image.

#### 2.5.2. Peak Signal-to-Noise Ratio (PSNR)

Derived directly from the MSE, the Peak Signal-to-Noise Ratio evaluates the ratio between the maximum possible power of the radiometric signal and the corrupting noise affecting its representation. It is compressed via a logarithmic scale to account for wide dynamic ranges and is defined as Equation (10).

(10) $$\mathrm{PSNR}=10\;\cdot \;\log_{10}\left(\frac{{\mathrm{MAX}}_{Y}^{2}}{\mathrm{MSE}}\right)$$

where ${\mathrm{MAX}}_{Y}$ represents the maximum valid pixel intensity value within the bit-depth of the sensor (e.g., 255 for standard 8-bit representations, or the maximum reflectance value for scaled 16-bit satellite products). Higher PSNR values, expressed in decibels (dB), represent cleaner, less noisy image reconstructions.

#### 2.5.3. Structural Similarity Index (SSIM)

To overcome the spatial limitations of MSE and PSNR, the Structural Similarity Index incorporates human visual system characteristics by assessing image patches based on three independent components: luminance (*l*), contrast (*c*), and structure (*s*). The overall index is calculated as Equation (11).

(11) $$\mathrm{SSIM}\left(Y,\hat{Y}\right)={\left[l\left(Y,\hat{Y}\right)\right]}^{\alpha }\;\cdot \;{\left[c\left(Y,\hat{Y}\right)\right]}^{\beta }\;\cdot \;{\left[s\left(Y,\hat{Y}\right)\right]}^{\gamma }$$

When setting the weighting exponents to unity (α=β=γ=1), the formula simplifies into local windows to Equation (12).

(12) $$\mathrm{SSIM}\left(Y,\hat{Y}\right)=\frac{\left(2\mu_{Y}\mu_{\hat{Y}}+C_{1}\right)\left(2\sigma_{Y\hat{Y}}+C_{2}\right)}{\left(\mu_{Y}^{2}+\mu_{\hat{Y}}^{2}+C_{1}\right)\left(\sigma_{Y}^{2}+\sigma_{\hat{Y}}^{2}+C_{2}\right)},$$

where $\mu_{Y}$ and $\mu_{\hat{Y}}$ represent the local mean intensities, $\sigma_{Y}^{2}$ and $\sigma_{\hat{Y}}^{2}$ are the local variances, and $\sigma_{Y\hat{Y}}$ is the cross-covariance. The terms $C_{1}$ and $C_{2}$ are small stabilization constants used to avoid division by zero. SSIM yields values bounded within [−1,1], where a score of 1.0 indicates structural identity. This is particularly vital in remote sensing to verify that geological boundaries, agricultural rows, and urban infrastructures preserve their geometric shapes.

#### 2.5.4. Learned Perceptual Image Patch Similarity (LPIPS)

Traditional metrics often penalize high-frequency textures generated by adversarial networks (such as ESRGAN) if those details do not align perfectly pixel-by-pixel, even when they appear highly realistic. To bridge this perceptual gap, LPIPS [21,28] leverages the deep features of a pre-trained convolutional neural network, such as VGG-16 [29], as a perceptual metric. Let ${\hat{y}}^{l},y^{l}\in R^{H_{l}\times W_{l}\times C_{l}}$ be the feature map tensor extracted from the *l*-th layer of the network for images $\hat{Y}$ and *Y*, respectively. The features are normalized along the channel dimension, scaled by a layer-specific weight vector $w^{l}$, and the $L_{2}$ distance is computed over all spatial dimensions (Equation (13)):

(13) $$\mathrm{LPIPS}\left(Y,\hat{Y}\right)=\sum_{l}\frac{1}{H_{l}W_{l}}\sum_{i,j}{∥w^{l}⊙\left(y_{i,j}^{l}-{\hat{y}}_{i,j}^{l}\right)∥}_{2}^{2},$$

where ⊙ denotes the Hadamard element-wise product. LPIPS values approach 0 as the perceptual distance decreases, making lower scores indicative of higher visual realism.

#### 2.5.5. Deep Image Structure and Texture Similarity (DISTS)

While LPIPS is highly sensitive to structural distortions, it can over-penalize minor spatial misalignments or shifts in stochastic textures, such as forest canopies, water bodies, or soil roughness patterns. DISTS [22] resolves this by unifying structural and texture similarity using an optimization model derived from deep neural activations.

Using deep feature maps from multiple layers, DISTS calculates global and local statistical features, computing a score based on a modification of the SSIM formulation applied directly to neural feature channels (Equation (14)):

(14) $$\mathrm{DISTS}\left(Y,\hat{Y}\right)=1-\sum_{l=0}^{L}\sum_{c=1}^{C_{l}}\left(w_{l,c}\;\cdot \;\frac{2\mu_{y^{\left(l,c\right)}}\mu_{{\hat{y}}^{\left(l,c\right)}}+C_{1}}{\mu_{y^{\left(l,c\right)}}^{2}+\mu_{{\hat{y}}^{\left(l,c\right)}}^{2}+C_{1}}\;\cdot \;\frac{2\sigma_{y^{\left(l,c\right)}{\hat{y}}^{\left(l,c\right)}}+C_{2}}{\sigma_{y^{\left(l,c\right)}}^{2}+\sigma_{{\hat{y}}^{\left(l,c\right)}}^{2}+C_{2}}\right)$$

where μ and σ represent the mean and variance of the activation maps for channel *c* at layer *l*, and $w_{l,c}$ denotes positive learnable weights that regulate the importance of structural vs. textural features. DISTS scores are bounded between 0 and 1, where a value close to 0 signifies that the synthesized satellite textures exhibit the same statistical properties and distribution as the high-resolution Sentinel-2 targets.

## 3. Experimental Methodology

### 3.1. Models Training

For models training, an approach based on transfer learning was employed; pretrained versions of the SwinIR and ESRGAN architectures were fine-tuned, using the created dataset as an information source. This methodology allowed the use of feature extraction and image reconstruction capabilities previously learned from large datasets, thus, the computational requirements for training from scratch were reduced.

#### 3.1.1. Swin Transformer for Image Restoration

For the training of the model based on the SwinIR architecture, the pretrained version for classic super-resolution with a ×3 scale factor was used. To analyze different levels of model adaptation, two training configurations were proposed. In the first configuration (Training 1), only the output convolution of the deep feature extraction module and the layers of the reconstruction module were trainable, while in the following configuration (Training 2), in addition to the layers specified in configuration 1, the last of the six blocks in the deep feature extraction module was also trainable.

The location of the blocks mentioned in both configurations is shown in Figure 1.

The training was carried out using the KAIR repository developed by Kai Zhang [30]. It provides inference and training algorithms and allows uploading pretrained models, modifying hyperparameters, and selecting which layers of the architecture will be trainable. In this framework, the created Landsat–Sentinel dataset was assigned as the data source for supervised training. In general, the hyperparameters were kept at the default values of the pretrained model. Only the batch size was modified, from 32 to 8, in order to reduce computational requirements.

#### 3.1.2. Enhanced Super-Resolution Generative Adversarial Network

For training the ESRGAN-based model, a pretrained super-resolution model with a ×4 scaling factor was used, since this architecture does not provide pretrained models with an ×3 scaling factor. However, the upsampling layers were modified to adapt the model to a ×3 scaling factor, corresponding to the relationship between the images used in this work.

Similar to the training strategy used for the SwinIR architecture, two training configurations were defined for the ESRGAN model to analyze the effect of different levels of model adaptation. The first configuration consisted of a partial fine-tuning that included only the reconstruction layers, while the second involved a deeper adaptation by allowing the last blocks of the feature extraction module to be trainable. To ensure a comparable adaptation depth between both models, fine-tuning was performed on a similar proportion of the feature extraction module, corresponding to 4 of the 23 feature extraction blocks in ESRGAN. The trainable layers for the two training configurations of the ESRGAN architecture are shown in Figure 2.

A similar training procedure was followed for the ESRGAN-based model using the BasicSR framework proposed in [31]. The same Landsat–Sentinel dataset was employed for supervised training, while the hyperparameters were kept at the default values of the pretrained model, except for the batch size, which was reduced to 8. Additionally, architectural modifications were performed to adapt the reconstruction module from ×4 to ×3 super-resolution.

After training both models, inference was performed on the 500 low-resolution images of the dataset using the fine-tuned models, their corresponding pretrained versions, and a bicubic interpolation model. The generated outputs were subsequently used for visual evaluation and for the computation of the selected metrics by comparing the reconstructed images with the high-resolution Sentinel images used as reference.

### 3.2. Friedman Test

Before the Friedman test, the D’Agostino–Pearson omnibus test was applied to verify the normality assumption for each metric and model combination. As shown in Table 1, the K^2^ statistics are large across all combinations, with *p*-values ranging from $3.13\times 10^{-4}$ (PSNR/SwinIR_T1, the least extreme case) to values below $10^{-260}$ for MSE-based distributions. All *p*-values fall well below the significance threshold of α=0.05, providing strong statistical evidence to reject the null hypothesis of normality across all metric–model combinations. Given that the independence assumption was also violated, since all models were evaluated on the same 500 test images, the use of parametric ANOVA was discarded, and the Friedman test was adopted as the appropriate non-parametric alternative for repeated-measures comparison. Given that the independence assumption was not accomplished, since all models were evaluated on the same 500 test images, the use of parametric ANOVA was discarded, and the Friedman test was adopted as the appropriate non-parametric alternative for repeated-measures comparison [32,33].

To determine whether there are statistically significant differences between the evaluated models in terms of their performance metrics, an analysis of variance (ANOVA) was conducted. This analysis allows us to evaluate whether the observed variations in the metrics can be attributed to the effect of the model used or, conversely, to the inherent variability of the data.

Parametric ANOVA is based on three fundamental assumptions. First, the normality assumption states that the observations within each group are drawn from approximately normal populations. Second, the homogeneity of variances assumption requires that the dispersion of the metrics be similar across the different evaluated models. Finally, the independence assumption states that each observation must be statistically independent of the others [33].

The assumptions required for parametric ANOVA were evaluated prior to statistical analysis. When these assumptions were not satisfied, the Friedman test was employed as a non-parametric alternative for the comparison of related groups [33].

The Friedman test is based on ranking the performance of each model across the different test samples. Let *n* denote the number of test images, each considered as a block, and *k* the number of evaluated models. Let $R_{ij}$ represent the rank of the *j*-th model in the *i*-th test image. The average rank of each model is defined as Equation (15).

(15) $${\bar{R}}_{j}=\frac{1}{n}\sum_{i=1}^{n}R_{ij}$$

The Friedman test statistic is given by Equation (16).

(16) $$\chi_{F}^{2}=\frac{12n}{k(k+1)}\sum_{j=1}^{k}{\bar{R}}_{j}^{2}-3n\left(k+1\right),$$

where *n* is the number of datasets and *k* is the number of models. Under the null hypothesis that all models perform equivalently, the statistic approximately follows a chi-square distribution with k−1 degrees of freedom (Equation (17)).

(17) $$\chi_{F}^{2}∼\chi_{k-1}^{2}$$

To verify the normality assumption for each performance metric and for each evaluated model, the D’Agostino–Pearson omnibus normality test was applied. This test is particularly suitable for moderate and large sample sizes and provides a global assessment of deviations from normality by simultaneously evaluating the skewness and kurtosis of the data distribution [34].

The sample skewness and kurtosis are computed as Equations (18) and (19) respectively.

(18) $$g_{1}=\frac{m_{3}}{m_{2}^{3/2}}$$

(19) $$g_{2}=\frac{m_{4}}{m_{2}^{2}}-3,$$

where the central moments are defined by Equation (20).

(20) $$m_{k}=\frac{1}{n}\sum_{i=1}^{n}{\left(x_{i}-\bar{x}\right)}^{k},$$

with *n* representing the sample size and $\bar{x}$ the sample mean.

The skewness and kurtosis statistics are then transformed into approximately standard normal variables, denoted by $Z_{1}$ and $Z_{2}$, respectively. These transformed variables are combined into the omnibus test statistic following Equation (21).

(21) $$K^{2}=Z_{1}^{2}+Z_{2}^{2}$$

Under the null hypothesis that the data follow a normal distribution, the statistic $K^{2}$ approximately follows a chi-square distribution with two degrees of freedom (Equation (22)).

(22) $$K^{2}∼\chi_{2}^{2}$$

A significance level of α=0.05 was adopted. Therefore, a *p*-value lower than 0.05 indicates sufficient evidence to reject the null hypothesis of normality.

For the metrics in which the analysis of variance rejected the null hypothesis, indicating the existence of statistically significant differences among the evaluated models, a post hoc Nemenyi test was subsequently performed to identify the specific pairs of models exhibiting significant differences. The Nemenyi procedure was selected because it is specifically designed for multiple pairwise comparisons following rank-based statistical tests while properly controlling the family-wise error rate. Furthermore, it is widely employed in comparative analyses involving multiple machine learning and image processing models evaluated over the same datasets.

The Friedman test was applied independently for each of the five evaluation metrics, treating each test image as a block and each model as a treatment. Table 2 summarises the resulting test statistics and *p*-values. For all five metrics, the Friedman statistic is large and the associated *p*-value equals zero to machine precision (p<0.0001), confirming that statistically significant differences exist among the ten evaluated models for every metric considered.

Since significant differences were detected for all metrics, a post hoc Nemenyi test was performed to identify which specific pairs of models differ significantly. The resulting *p*-value matrices are shown in Table 2. Cells with p<0.05 indicate statistically significant pairwise differences. The heatmaps reveal a consistent pattern: the two fine-tuned ESRGAN configurations (ESRGAN (train 1) and ESRGAN (train 2)) are significantly different from all baseline models (bicubic, ESRGAN pretrained, SwinIR pretrained) across all pixel-wise metrics (MSE, PSNR, SSIM). For perceptual metrics (LPIPS, DISTS), the distinction between fine-tuned and baseline models is equally significant, with ESRGAN (train 2) consistently occupying the lowest average rank. Notably, the SwinIR fine-tuned variants (train 1 and train 2) also differ significantly from their pretrained baseline, though the magnitude of improvement is smaller than that achieved by ESRGAN fine-tuning.

### 3.3. Results

The following analysis presents the quantitative evaluation of the super-resolution models across the 500 test images.

Figure 3 provides a visual comparison of super-resolution outputs for five representative test scenes covering diverse land cover types including agricultural fields, forest areas, urban settlements, and bare soil. Several observations are consistent across scenes. Bicubic interpolation produces smooth outputs that preserve low-frequency structure but blur fine spatial details such as field boundaries, road networks, and riparian vegetation edges. The pretrained ESRGAN and SwinIR models introduce visually sharper textures but exhibit hallucinated artifacts, particularly in high-contrast urban and bare-soil areas, which explains their degraded pixel-wise scores despite improved perceptual ratings. After domain-specific fine-tuning, both ESRGAN (train 2) and SwinIR (train 2) recover geographic detail more faithfully, with edge preservation and texture coherence clearly improved relative to their pretrained counterparts. ESRGAN (train 2) produces the sharpest and most geographically coherent outputs overall, though occasional GAN-induced artifacts remain visible in high-contrast urban patches, consistent with the known limitations of adversarial training. Table 3 includes the description of images used in the quantitative comparison of Figure 3, including file name randomly generated for the dataset, latitude, longitude, and Sentinel and Landsat acquisition dates, respectively.

The quantitative evaluation was performed on the 500 independent test pairs, which constitute the held-out test subset of the complete dataset of 3500 paired Landsat–Sentinel-2 patches. The same acquisition and filtering criteria described above were applied during the construction of the complete dataset.

**Table 3:** Description of five image sample of the dataset used in Figure 4.

Image	Filename	Latitude	Longitude	Sentinel Date	Landsat Date
1	ioVZGhfs0I	29.705289	114.029575	19 October 2022	20 October 2022
2	v1RlGuNEhp	28.040386	−80.613133	15 January 2023	19 January 2023
3	35m3spagiF	14.580515	100.984893	7 March 2022	4 March 2022
4	4sqkdYzBdd	17.226054	106.788034	15 January 2023	13 January 2023
5	ewCtqwQpiN	16.522921	120.340250	30 January 2023	30 January 2023

Figure 5 presents the 95% confidence intervals of the evaluation metrics across the evaluated models, providing an aggregate comparison of their performance over the complete independent test subset. For the MSE, PSNR, and SSIM metrics, the pretrained SwinIR and ESRGAN models exhibit confidence intervals that largely overlap with those obtained using bicubic interpolation. This substantial overlap indicates that the performance of the pretrained models is statistically comparable to that of bicubic interpolation for these metrics. In contrast, for the perceptual metrics LPIPS and DISTS, a more pronounced separation between the confidence intervals is observed, suggesting more noticeable performance differences relative to the interpolation baseline.

In contrast, the fine-tuned models exhibit consistent improvements in the MSE, PSNR, and SSIM metrics, reflected by a greater separation of their confidence intervals from both bicubic interpolation and the pretrained models. This separation is particularly evident for the ESRGAN configurations, whose confidence intervals are clearly distinguished from the group formed by bicubic interpolation and the pretrained models. In the case of SwinIR, although fine-tuning also improves performance, the separation between the confidence intervals is less pronounced, and partial overlap with the pretrained models remains for some metrics, reflecting a more modest adaptation gain. For the LPIPS and DISTS metrics, the fine-tuned ESRGAN models also exhibit a greater separation of their confidence intervals relative to the pretrained version, indicating an improvement in the perceptual quality of the reconstructed images. For SwinIR, this behavior is clearly observed only for Training Configuration 2, whereas Training Configuration 1 remains closer to the pretrained model.

Overall, the fine-tuning process improves model performance with respect to both bicubic interpolation and the pretrained versions. Among all the evaluated configurations, ESRGAN (train 2) achieves the best overall performance, consistently obtaining the most favorable results across all five evaluation metrics (MSE, PSNR, SSIM, LPIPS, and DISTS). Furthermore, the confidence intervals associated with this configuration are clearly separated from those of the baseline models, visually reinforcing the consistency of its superior performance.

Nevertheless, when comparing the different training configurations, the confidence intervals exhibit partial overlap across several of the evaluated metrics. Although differences in the mean values can be visually observed, the presence of these overlaps prevents determining, based solely on graphical inspection, whether the observed differences are statistically significant. Consequently, confidence intervals provide a useful tool for identifying general trends in model performance but are not sufficient on their own to establish statistical significance. Therefore, this analysis must be complemented with a statistical comparison of the models. In this study, the non-parametric Friedman test, followed by the Nemenyi post hoc test, was employed to determine whether the observed differences among the models are statistically significant or can be attributed to the inherent variability of the data.

Figure 6 displays the full distribution of metric values across the 500 test images for each model. The boxplots reveal two complementary aspects of model behavior that cannot be captured by itself. First, the pretrained models and bicubic interpolation exhibit long upper tails in MSE, indicating that all three struggle with high-contrast scenes (e.g., urban areas, bare soil transitions) where pixel-level errors accumulate. Second, and more importantly, ESRGAN (train 2) not only achieves the lowest median MSE but also the most compact interquartile range, reflecting consistently reliable performance across all land cover types in the test set. For SSIM, all models show a left-skewed distribution concentrated near 1.0, but the fine-tuned ESRGAN configurations shift the median upward and reduce the frequency of low-SSIM outliers. For LPIPS and DISTS, the pretrained ESRGAN model already shows a lower median than bicubic, confirming the perceptual advantage of adversarial priors even before fine-tuning—a nuance that would be invisible in a mean-only comparison.

Since the Landsat and Sentinel-2 observations were paired with a maximum temporal difference of five days, we additionally assessed whether this temporal separation contribute to the reconstruction errors or not. The temporal distribution of the paired acquisitions is shown in Figure 7. The 35.2% of the pairs correspond to same-day acquisitions and 30.2% have a one-day temporal difference, so 65.4% of the test pairs have a temporal separation of one day or less, whereas 34.6% of the pairs have a separation between two and five days.

To assess the potential influence of temporal separation on model performance, the independent test pairs were grouped into three temporal-gap categories: 0 days, 1 day, and 2–5 days. The distributions of the metrics MSE, PSNR, SSIM, LPIPS, and DISTS for each model and temporal group are shown in Figure 8. The MSE distributions exhibit pronounced upper tails across the temporal groups, consistent with the sensitivity of this metric to large pixel-wise discrepancies. However, the distributions of the three temporal groups are comparable across the evaluated models and metrics. Also, a Kruskal–Wallis test was independently applied to each model and metric combination to determine if the metric distributions differed significantly according to temporal separation. The null hypothesis assumes that the distributions of a given metric are equivalent across the three temporal-gap groups. No statistically significant differences were detected for any of the 35 model–metric comparisons (p>0.05). The smallest obtained *p*-value was for DISTS with ESRGAN Train 1 (p=0.061). Hence, the observed performance does not show statistically significant variation associated with the temporal gap between the Landsat and Sentinel-2 acquisitions with the temporal range considered in this dataset.

Figure 9 presents the *p*-value matrices obtained from the Nemenyi post hoc test for each evaluation metric. Each cell corresponds to a pairwise comparison between two models, where values close to 1 (blue) indicate the absence of statistically significant differences, whereas values close to 0 (red) indicate statistically significant differences (*p* < 0.05).

For the MSE and PSNR metrics, models trained with the same fine-tuning depth do not exhibit statistically significant differences, regardless of the architecture employed. In particular, the obtained *p*-values were 0.133 for the comparison between SwinIR train 1 and ESRGAN train 1, and 0.999 for SwinIR train 2 and ESRGAN train 2. In contrast, comparisons between models trained with different fine-tuning depths reveal statistically significant differences, indicating that fine-tuning depth has a decisive influence on performance according to these pixel-wise metrics. Furthermore, the pretrained SwinIR and ESRGAN models do not differ significantly from bicubic interpolation, confirming that the pretrained models achieve performance statistically comparable to the interpolation baseline.

A different pattern is observed for the SSIM metric. In this case, statistically significant differences are found between SwinIR and ESRGAN even when both models are trained with the same fine-tuning depth, with *p*-values of 0.046 for train 1 and 0.025 for train 2. Moreover, SwinIR train 1 does not differ significantly from bicubic interpolation, yielding a *p*-value of 0.998. These results indicate that SSIM provides greater discrimination between architectures than MSE and PSNR.

The perceptual metrics LPIPS and DISTS exhibit the greatest degree of statistical separation among the evaluated models. Nearly all pairwise comparisons are statistically significant, with *p*-values close to zero, providing strong evidence against the null hypothesis of equal performance. The only exception corresponds to the comparison between SwinIR train 1 and bicubic interpolation, for which no statistically significant difference is detected. This result suggests that the first fine-tuning configuration of SwinIR is insufficient to produce perceptually detectable improvements over the interpolation baseline.

Overall, the Nemenyi test shows that the statistical differences significance between models depend on the evaluation metric. While MSE and PSNR primarily distinguish models according to their fine-tuning depth, SSIM further discriminates between the SwinIR and ESRGAN architectures. This discriminative capability is even more pronounced for the perceptual metrics LPIPS and DISTS, where nearly all pairwise comparisons are statistically significant. These findings are consistent with the confidence interval analysis and confirm that deeper fine-tuning, particularly for ESRGAN, produces statistically significant improvements across both pixel-wise and perceptual evaluation metrics.

## 4. Discussion

### 4.1. Discussion of Results

The experimental results reveal three fundamental insights about the application of super-resolution models to satellite imagery, each with direct implications for remote sensing practice. In all of them a notable dataset limitation arises from the operational differences between satellite missions. Due to the distinct orbital configurations, revisit cycles, and swath overlaps of Landsat and Sentinel-2, acquiring cross-sensor image pairs captured on the same date for the same region is practically unfeasible at scale. To construct a viable paired dataset, a maximum temporal window of up to 5 days was adopted, paired with the closest available acquisition dates. While this threshold minimizes major phenological shifts, short-term surface variations—such as changes in soil moisture, localized vegetation status, water body levels, or cloud cover and shadow dynamics—may introduce localized radiometrical or structural disparities between paired scenes. Consequently, these environmental dynamics represent an inherent baseline noise in the dataset, which can marginally influence pixel-level reconstruction evaluation metrics.

#### 4.1.1. Pretrained Models Do Not Improve Pixel-Wise Fidelity over Bicubic

One of the most relevant findings of this study is that the pretrained ESRGAN and SwinIR models failed to consistently improve radiometric fidelity over bicubic interpolation. Although both architectures were originally developed for natural-image super-resolution, their performance on satellite imagery indicates that the knowledge learned during pretraining does not transfer directly to this domain.

A plausible explanation is the presence of a domain adaptation gap between the natural-image datasets used for pretraining and the remote sensing imagery employed in this work. Models trained on datasets such as DIV2K and ImageNet learn representations optimized for RGB photographs, whereas Landsat and Sentinel-2 images exhibit distinct radiometric and spatial characteristics determined by sensor spectral responses, atmospheric effects, and the physical properties of surface reflectance. Consequently, the representations learned during pretraining are not fully suited to model the relationship between low- and high-resolution satellite images, which is consistent with the higher reconstruction errors observed in the pixel-wise evaluation metrics.

This behavior also helps explain why the pretrained models achieved better performance on perceptual metrics than on pixel-wise metrics. Both ESRGAN and SwinIR incorporate mechanisms designed to generate visually plausible textures, even when these textures do not correspond exactly to the reference image. As a result, the reconstructed images may appear sharper to human observers while still exhibiting radiometric discrepancies that degrade metrics such as MSE, PSNR, and, in some cases, SSIM.

Overall, these findings indicate that the representational capacity of a super-resolution model alone is insufficient to ensure successful transfer across domains. For satellite image super-resolution, effective domain adaptation is essential to preserve both radiometric fidelity and perceptual quality, highlighting the importance of fine-tuning pretrained models using domain-specific remote sensing data before their practical application.

#### 4.1.2. Fine-Tuning Reverses Degradation and Improves All Metrics

Domain-specific fine-tuning using the paired Landsat–Sentinel dataset consistently improved the performance of both super-resolution models, demonstrating that domain adaptation is an essential step for satellite image super-resolution. Unlike their pretrained counterparts, the fine-tuned models preserved both radiometric fidelity and perceptual quality more effectively, indicating that the representations learned during pretraining can be successfully adapted when exposed to domain-specific remote sensing data.

Among all the evaluated configurations, ESRGAN train 2 achieved the best overall performance by obtaining the most favorable results across all five evaluation metrics. This behavior suggests that deeper fine-tuning adapts not only the reconstruction layers but also the internal feature representations learned by the network, thereby reducing the discrepancy between the natural-image domain and satellite imagery. Nevertheless, even the shallower fine-tuning configuration produced noticeable improvements over the pretrained models, indicating that partial parameter adaptation is sufficient to capture domain-specific characteristics of Landsat and Sentinel-2 imagery without retraining the entire network. The differences observed between the two architectures further indicate that the effectiveness of fine-tuning depends on the underlying model design. While both ESRGAN fine-tuning configurations achieved statistically significant improvements over the baseline models across all evaluated metrics, SwinIR required the deeper fine-tuning configuration to achieve consistent improvements in the perceptual metrics, whereas the shallower configuration remained closer to the behavior of the pretrained model. This suggests that transformer-based architectures require more extensive parameter adaptation to fully exploit their representational capacity for satellite image super-resolution.

From a practical perspective, these findings indicate that substantial performance improvements can be achieved using a relatively small paired training dataset (3000 image pairs), thereby avoiding the high computational cost associated with training a super-resolution model from scratch. At the same time, the results demonstrate that the fine-tuning strategy should be tailored to the underlying architecture, as the extent of parameter updating directly influences the model’s ability to generalize to the satellite imagery domain.

#### 4.1.3. ESRGAN Outperforms SwinIR After Fine-Tuning

A consistent pattern emerges in the structural and perceptual metrics: fine-tuned ESRGAN variants outperform their SwinIR counterparts on SSIM (*p* = 0.046 for train 1, *p* = 0.025 for train 2) and on LPIPS/DISTS, whereas on MSE and PSNR the two architectures are statistically indistinguishable at equal fine-tuning depth (*p* = 0.133 for train 1, *p* = 0.999 for train 2). The confidence intervals for the trained ESRGAN models show greater separation from the pretrained baseline compared to the SwinIR variants, particularly for perceptual metrics. Two factors explain this difference. First, the adversarial training objective of ESRGAN explicitly encourages the generation of high-frequency textures, which are precisely the details lost during spatial degradation. Second, the RRDB backbone without batch normalisation is more stable for domain adaptation than the transformer-based architecture when the target domain differs significantly from the pre-training domain. However, this perceptual advantage comes with a trade-off: ESRGAN (train 2) exhibits occasional GAN-induced artefacts in high-contrast urban patches, as noted in the qualitative analysis (Figure 3), whereas SwinIR outputs are more conservative and artefact-free.

The superior stability of the RRDB backbone under domain shift can be attributed to its dense connection structure, which promotes feature reuse across multiple scales without requiring batch statistics (eliminated by the removal of Batch Normalisation layers). In contrast, the Shifted Window attention mechanism of SwinIR, while highly effective for natural image restoration, relies on learned relative position biases that encode spatial relationships specific to the pre-training domain. When fine-tuned on satellite patches—which exhibit fundamentally different spatial autocorrelation structures compared to natural photographs — these position biases require deeper adaptation than the shallow configurations tested here allow. This interpretation is consistent with the observation that SwinIR Training Configuration 2 exhibits a greater separation from the pretrained baseline than Configuration 1, suggesting that further unfreezing of attention layers could narrow the gap with ESRGAN in future work.

#### 4.1.4. Perceptual and Pixel-Wise Metrics Diverge

The divergence between traditional and perceptual metrics observed in the pretrained models is worth emphasising. While pretrained models perform comparably to bicubic on pixel-wise metrics (MSE, PSNR, SSIM), they substantially improve perceptual quality (LPIPS, DISTS) as evidenced by the more pronounced separation of confidence intervals for these metrics. This means that a model can generate images that are perceptually more realistic (sharper, better textures) while simultaneously maintaining comparable pixel-level errors relative to the reference. For satellite monitoring applications where both radiometric fidelity and visual interpretability matter, relying exclusively on either metric family would be misleading. Our hybrid evaluation approach, combining MSE/PSNR/SSIM with LPIPS/DISTS, provides a more complete assessment.

This divergence has practical consequences for operational remote sensing. In applications such as visual land cover mapping or image interpretation by human analysts, perceptual quality (LPIPS, DISTS) may be the dominant criterion; in applications such as spectral change detection, vegetation index computation, or radiometric calibration, pixel-wise fidelity (MSE, PSNR) is critical and must not be sacrificed. The results presented here demonstrate that the fine-tuned ESRGAN (train 2) is the model that most consistently optimises both criteria, making it the recommended choice for operational deployment in contexts where both types of fidelity matter. For applications where artefact-free output is paramount—such as automated classification pipelines sensitive to GAN-induced noise—SwinIR (train 2) represents a more conservative but reliable alternative.

### 4.2. Comparison with State-of-the-Art Algorithms

Our results compare favourably with recent advances in satellite image super-resolution. Salgueiro Romero et al. [35] achieved PSNR values around 28–30 dB for Sentinel-2 super-resolution using ESRGAN with WorldView-2 as HR reference, considerably lower than our best results. The difference likely stems from three factors: (i) their scale factor of 5 (10 m to 2 m) is more challenging than our factor of 3, (ii) their HR reference (WorldView-2) is not radiometrically harmonised with Sentinel-2, and (iii) their training dataset was limited to two geographic locations (New York and Cairo), while our dataset spans latitudes −30° to 30° globally.

Huo et al. [14] proposed STGAN, a hybrid CNN-Swin Transformer GAN for remote sensing super-resolution, reporting PSNR = 31.42 dB and SSIM = 0.841 on public datasets. Our best model surpasses these values despite operating on a more challenging sensor pairing (Landsat to Sentinel-2 rather than downsampled high-resolution images from a single sensor).

More directly comparable is the work of Massarelli [36], who applied ESRGAN to Sentinel-2 super-resolution using WorldView-2 as reference, reporting PSNR ≈ 34.8 dB. Our best result is slightly higher, which we attribute to our careful fine-tuning strategy and the use of paired Landsat-Sentinel data that preserves the exact degradation kernel of the target application.

A key differentiator of our work is the systematic comparison of shallow vs. deep fine-tuning, which has not been quantitatively characterised in previous satellite super-resolution literature. Most prior studies either train from scratch or freeze the entire pretrained backbone; our results demonstrate that intermediate levels of unfreezing yield optimal performance, balancing adaptation depth against overfitting risk.

It is also worth noting that none of the studies cited above employ non-parametric statistical validation to confirm the significance of their reported differences. The use of the Friedman test with Nemenyi post hoc analysis in this work represents a methodological advance over simple mean comparisons, ensuring that the reported improvements are not attributable to dataset variability. This distinction is particularly relevant given the high variance observed in MSE across land cover types (shown in the boxplots of Figure 6), which could easily produce misleading mean-based rankings without formal hypothesis testing.

### 4.3. Novelty of the Research

The contributions of this work extend beyond the immediate quantitative improvements. Four aspects constitute genuine novelty:

According to the state-of-the-art review, this is the first study to quantitatively compare shallow vs. deep fine-tuning strategies for satellite super-resolution. The finding that deeper fine-tuning (unfreezing feature extraction blocks) outperforms shallow fine-tuning (reconstruction layers only), while both outperform pretrained baselines, provides practical guidance for future practitioners.

We provide clear empirical evidence that off-the-shelf natural-image super-resolution models can degrade pixel-wise fidelity when applied directly to satellite data. This counter-intuitive result serves as a cautionary note for researchers who might assume that better perceptual quality implies better reconstruction fidelity.

The paired Landsat-Sentinel dataset of 3500 patches with rigorous cloud, water, and spectral variability filters, along with the fully automated GEE acquisition pipeline, constitutes a reproducible resource for the community. The diversity of geographic coverage (latitudes −30° to 30°) and land cover types (forest, desert, urban, water, agriculture, paramo) ensures broad applicability.

Finally, by combining traditional metrics (MSE, PSNR, SSIM) with perceptual metrics (LPIPS, DISTS) and statistical validation (Friedman test with Nemenyi post hoc), we provide a multidimensional assessment that reveals trade-offs invisible to any single metric. The finding that pretrained models improve LPIPS while degrading PSNR would have been obscured by using either metric family alone.

### 4.4. Potential Environmental Applications

The proposed downscaling framework has potential applications beyond conventional satellite image enhancement. In particular, it could contribute to improving the spatial resolution of global environmental products derived from Earth Observation data, where coarse spatial resolution often limits local-scale analyzes and decision making [13]. These finer-scale patterns often correspond to environmental and territorial changes of considerable significance, where timely detection and effective management are essential to support environmental conservation, sustainable land-use planning, natural resource management, disaster risk reduction, and evidence-based decision-making [37].

Although deep learning-based super-resolution has traditionally been evaluated using image quality metrics, an emerging body of research highlights its broader potential for environmental applications, including the downscaling of environmental indicators, meteorological variables, hydrological products, and long-term Earth observation datasets [37,38]. As highlighted by [37], environmental information is frequently available at spatial scales that do not match the scales required for environmental assessment, management, and decision-making. Downscaling has therefore become a fundamental strategy for bridging this mismatch by transforming coarse-resolution environmental information into finer-scale products better suited to local analyses and applications.

Improving the spatial resolution of remotely sensed environmental products could provide a more detailed understanding of environmental processes whose spatial heterogeneity is better characterized at finer spatial resolutions. For example, studies on hydrogeomorphological processes, including erosion, delta progradation, meander migration, and floodplain evolution, frequently require high-spatial-resolution imagery to accurately delineate geomorphic features and detect subtle landscape changes [39,40]. Consequently, recent studies have relied on Sentinel-2 optical imagery, often complemented by Sentinel-1 SAR data, to characterize these processes [39,40]. Although these datasets substantially improve the spatial representation of hydrogeomorphological features, their relatively short observation record limits the investigation of long-term environmental dynamics. In contrast, the Landsat archive provides more than four decades of continuous observations, offering an unparalleled temporal record of landscape evolution [10,11]. Therefore, applying the proposed downscaling framework to Landsat images could bridge the gap between spatial and temporal resolution, allowing retrospective analyzes of long-term geomorphological evolution while preserving the spatial detail required to identify fine-scale environmental changes [10,39,40,41,42].

The potential benefits of this approach extend beyond hydrogeomorphological applications. Several widely used global environmental products, including the Global Surface Water dataset [4] and the Global Forest Change dataset [5], were developed from the Landsat archive because its unparalleled temporal continuity enables the reconstruction of multi-decadal environmental dynamics at the global scale [10]. Although these products have become fundamental resources for environmental monitoring, their spatial resolution is inherently constrained by the native 30-m resolution of the Landsat archive [10]. Applying robust deep learning-based downscaling techniques to historical Landsat imagery could help alleviate this long-standing trade-off between spatial and temporal resolution, enabling finer-scale analyzes while preserving the historical perspective that has made these datasets indispensable for global environmental change studies.

### 4.5. Future Work

Despite the strengths of this study, several limitations and research directions warrant future investigation.

The current work uses only RGB bands. While this choice simplifies the problem and enables direct comparison with natural-image super-resolution models, it discards valuable spectral information present in Landsat and Sentinel-2, particularly the near-infrared (NIR) and short-wave infrared (SWIR) bands. These bands are critical for vegetation monitoring, water body detection, and soil moisture estimation. Future work will extend the framework to multi-spectral inputs, modifying the first convolutional layer of SwinIR and ESRGAN to accept 4–6 channels.

The results presented here suggest that SwinIR’s performance under domain shift is sensitive to the number of transformer blocks unfrozen during fine-tuning. Training Configuration 2 already yields substantial improvement relative to the pretrained baseline, but still trails the fine-tuned ESRGAN by a considerable margin. A natural extension is to systematically evaluate configurations that unfreeze 2, 3, or all 6 RSTB blocks, potentially combined with a lower learning rate for the deeper layers (layer-wise learning rate decay). This analysis could determine whether SwinIR’s conservative reconstruction style is an inherent architectural limitation or simply a consequence of insufficient fine-tuning depth.

The paired dataset constructed in this work exploits the temporal overlap between Landsat and Sentinel-2 (2015–present). A promising extension is to leverage the full multi-decadal Landsat archive (1972–present) by training a temporally aware super-resolution model that fuses multiple low-resolution Landsat acquisitions to produce a single high-resolution output consistent with Sentinel-2 radiometry. This would effectively extend the Sentinel-2-equivalent record back to the 1970s, enabling long-term land cover change analyses at 10 m resolution.

The framework developed here is not inherently limited to the Landsat–Sentinel-2 pairing. The same transfer learning approach could be applied to other sensor combinations of practical interest, such as MODIS-to-Landsat (scale factor ×16, relevant for time-series analysis), Sentinel-2-to-WorldView (scale factor ×5, relevant for urban monitoring), or PlanetScope-to-aerial imagery. Each pairing introduces different radiometric and geometric challenges, and the comparative fine-tuning analysis developed here provides a methodological template for evaluating adaptation depth in each case.

The ultimate motivation for Landsat-to-Sentinel super-resolution is to enable consistent long-term land cover monitoring. A direct validation of practical utility would be to integrate the fine-tuned ESRGAN (train 2) model into a change detection pipeline—for example, computing the Normalised Difference Vegetation Index (NDVI) or urban expansion metrics on super-resolved Landsat images and comparing the results against those obtained directly from Sentinel-2 acquisitions. Such an end-to-end evaluation would complement the patch-level metrics reported here with application-level evidence of value.

Both SwinIR and ESRGAN are computationally demanding models designed for offline processing. For operational deployment in resource-constrained environments, such as onboard satellite processing units or edge computing nodes in environmental monitoring networks, lightweight alternatives such as knowledge distillation from the fine-tuned ESRGAN (train 2) into a compact student network could be explored. The paired dataset and evaluation framework developed in this work provide the necessary infrastructure for training and benchmarking such models.

The stochastic nature of deep learning training may introduce variability in the obtained model parameters and performance. In this study, the random seeds used during training were not explicitly recorded, hence, the variability associated with different random seeds was not quantified. Although repeated training with multiple random seeds could provide an additional assessment of model robustness, this analysis is beyond the scope of this study. To facilitate reproducibility and enable future multi-seed analyses, the complete training code is publicly available on GitHub (CLI 2.39.2), while the paired Landsat–Sentinel-2 dataset and the trained model weights are available on Hugging Face. Future work will consider repeated training with different random seeds to quantify the variability of the reported results and further assess the robustness of the proposed approach.

## 5. Conclusions

This study investigated the effectiveness of transfer learning for adapting state-of-the-art super-resolution architectures, specifically SwinIR and ESRGAN, to the task of Landsat-to-Sentinel-2 spatial downscaling with a ×3 scale factor. Based on the experimental evaluation and statistical analysis, the following conclusions can be drawn.

The pretrained models failed to outperform simple bicubic interpolation in pixel-wise metrics, demonstrating that the direct transfer of models trained on natural-image datasets is insufficient for satellite image super-resolution. This behavior highlights the existence of a domain adaptation gap, in which the representations learned during pretraining are not fully compatible with the radiometric and spatial characteristics of Earth observation imagery. Consequently, applying pretrained super-resolution models without domain-specific adaptation may compromise radiometric fidelity rather than improve it.

Domain-specific fine-tuning effectively reduced this domain adaptation gap. Using a relatively small paired Landsat–Sentinel-2 training dataset, both architectures achieved substantial improvements over their pretrained counterparts and the bicubic interpolation baseline. These findings demonstrate that the limitations observed in pretrained models are not inherent to the architectures themselves, but rather the consequence of insufficient adaptation to the target domain. Moreover, they show that significant performance gains can be achieved through transfer learning without incurring the high computational cost and large training datasets typically required to train a super-resolution model from scratch.

The depth of fine-tuning played a key role in the adaptation process. For both architectures, updating a larger portion of the network consistently produced better results than restricting optimization to the reconstruction layers alone, indicating that adapting intermediate feature representations is necessary to capture the statistical characteristics of satellite imagery. However, the response to fine-tuning differed between architectures, demonstrating that different models require different degrees of parameter adaptation to achieve their full potential.

Among all the evaluated configurations, ESRGAN train 2 achieved the best overall performance, providing the best balance between radiometric fidelity and perceptual quality. This result demonstrates that the combination of the RRDB backbone, the composite loss function, and deeper fine-tuning enables more effective adaptation to the satellite image domain. In contrast, although SwinIR also benefited from fine-tuning, its improvements were more moderate, particularly for the perceptual metrics, suggesting that transformer-based architectures require more extensive adaptation of their internal representations when transferred from natural-image restoration to remote sensing applications.

The divergent behavior observed between pixel-wise and perceptual metrics demonstrates that neither family of metrics alone is sufficient to assess super-resolution quality in satellite imagery. Models capable of generating visually realistic reconstructions do not necessarily preserve radiometric fidelity, highlighting the need to evaluate both aspects simultaneously. In this context, the hybrid evaluation framework proposed in this work, combining pixel-wise and perceptual metrics together with non-parametric statistical hypothesis testing, provides a more comprehensive and robust methodology for comparing satellite image super-resolution models.

The statistical validation based on the Friedman and Nemenyi tests confirmed that the observed differences among the evaluated models cannot be attributed to random dataset variability. Incorporating rigorous statistical analysis into the evaluation process therefore represents an important methodological contribution and provides a more reliable evaluation framework than that commonly adopted in satellite image super- resolution studies.

Finally, the proposed framework demonstrates that transfer learning combined with domain-specific fine-tuning is an effective and computationally efficient strategy for enhancing the spatial resolution of long-term Earth observation archives while preserving their temporal continuity. Beyond the Landsat–Sentinel-2 application considered in this study, the proposed methodology can be extended to other remote sensing products, facilitating the development of high-performance super-resolution models with moderate data and computational requirements while supporting higher-resolution environmental analyses without sacrificing the long-term observational records required for monitoring environmental change.

## Figures and Tables

**Figure 1:**
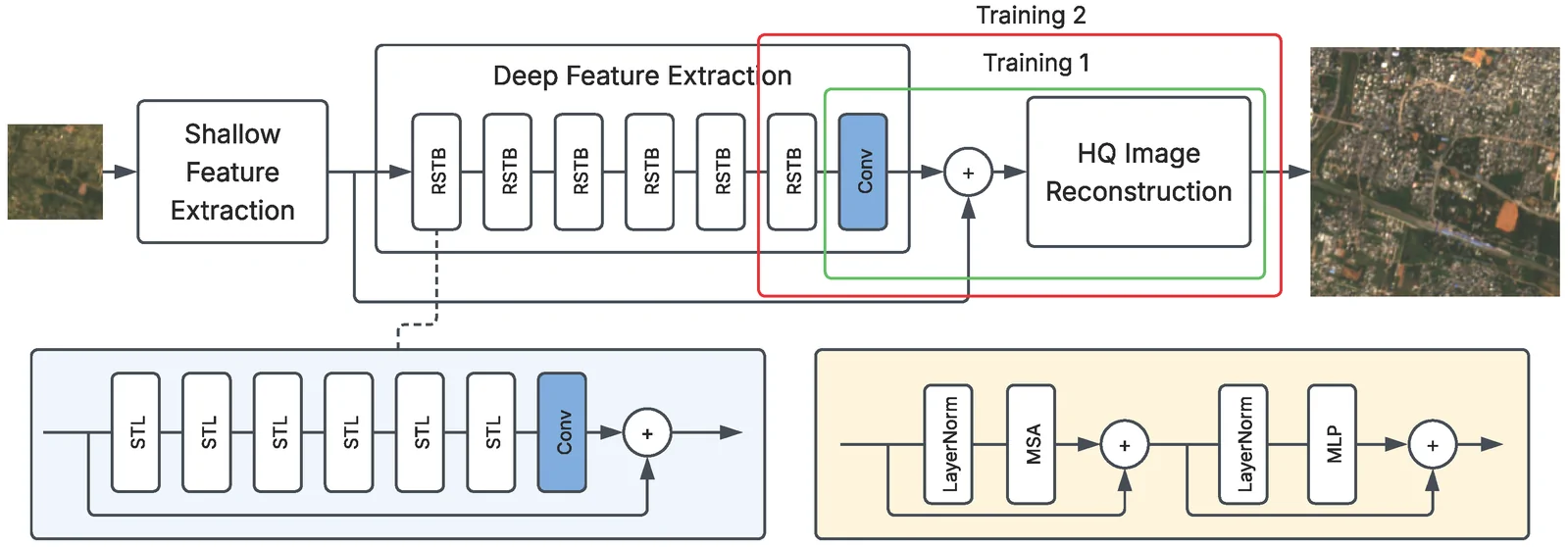
Location of the modified layers in the SwinIR architecture, illustrating the Landsat low-resolution input and the Sentinel-2-like high-resolution output used in the satellite image downscaling task (Adapted from [16]).

**Figure 2:**
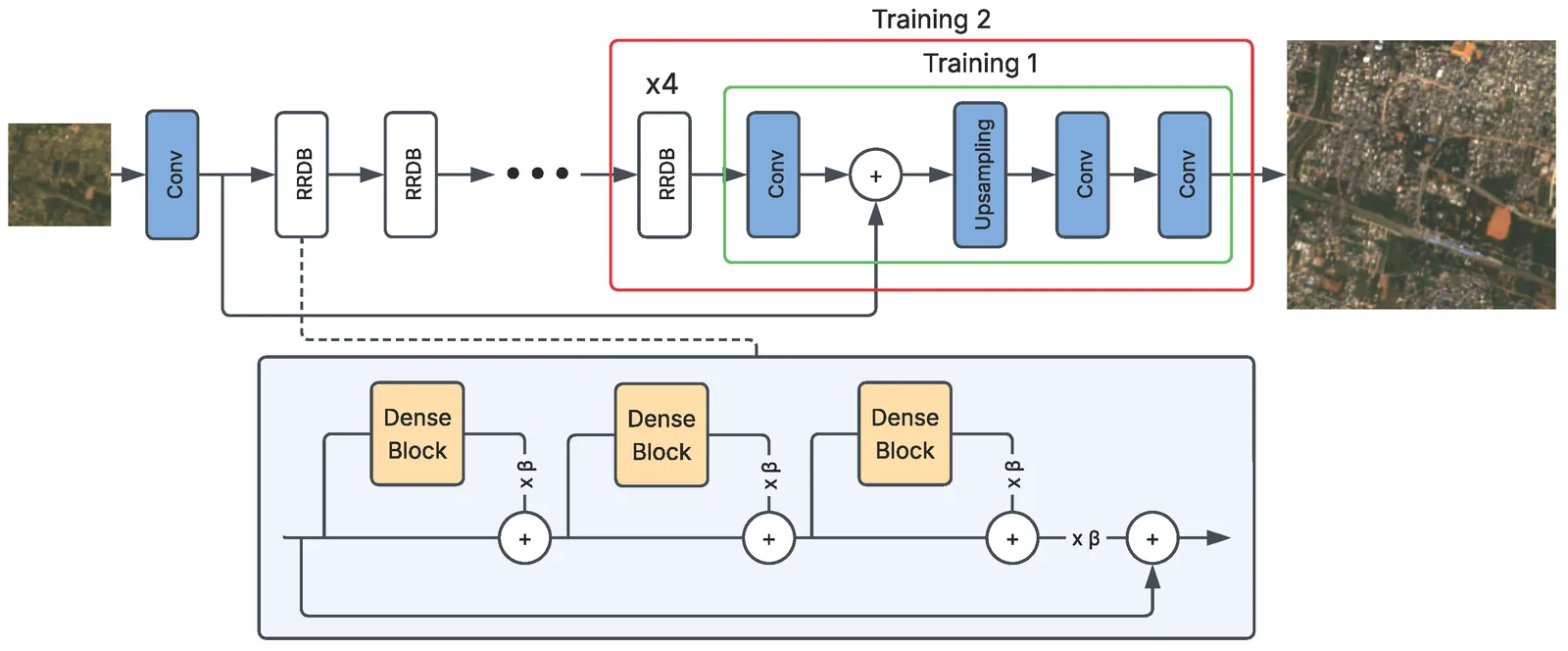
Location of the modified layers in the ESRGAN architecture training, illustrating the Landsat low-resolution input and the Sentinel-2-like high-resolution output used in the satellite image downscaling task (Adapted from [26]).

**Figure 3:**
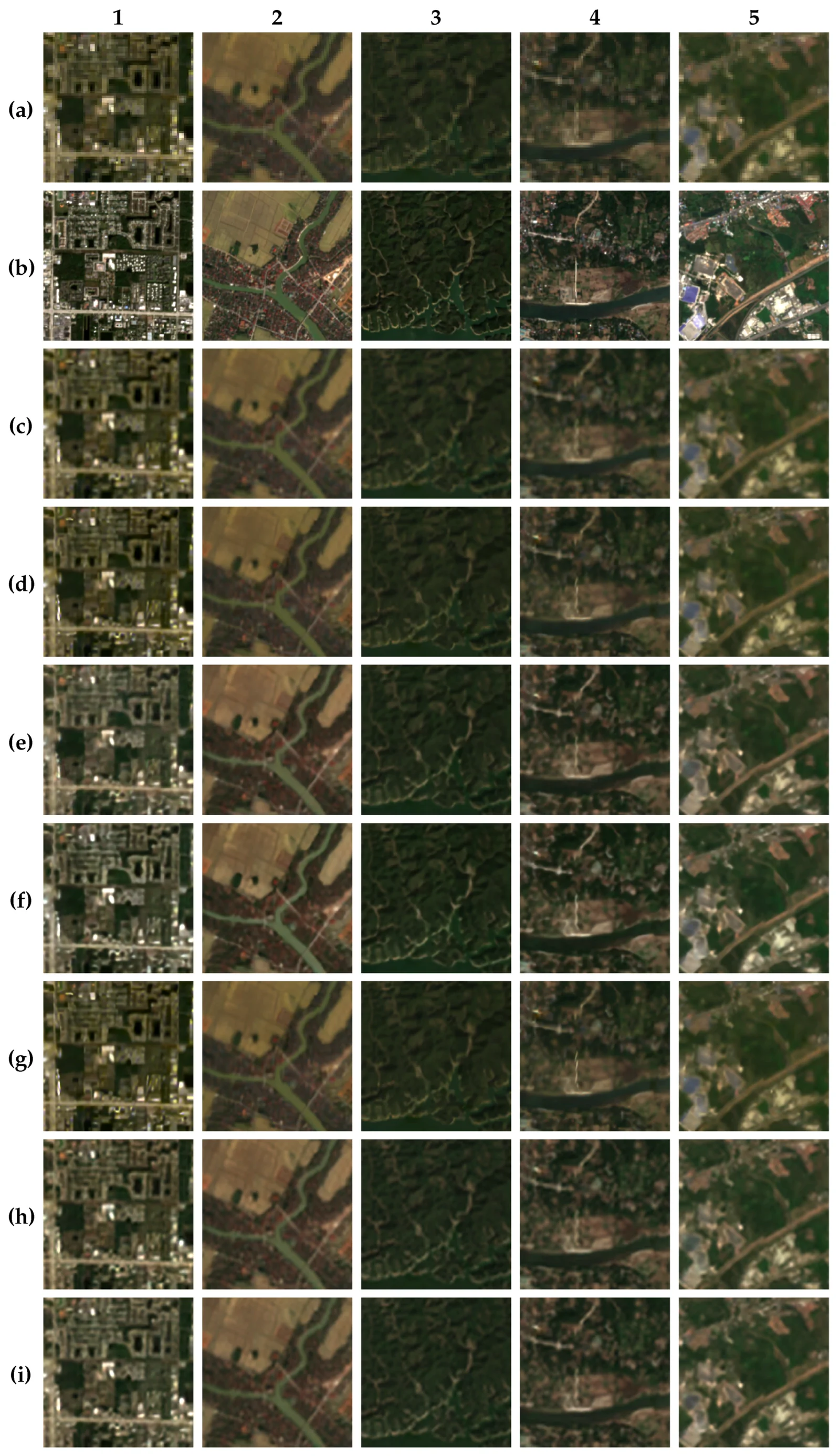
Qualitative comparison of super-resolution results on sample images 1–5 from the dataset. (**a**) Low-resolution inputs, (**b**) ground-truth high-resolution images, (**c**) bicubic interpolation, (**d**) ESRGAN pretrained model, (**e**) ESRGAN (train 1), (**f**) ESRGAN (train 2), (**g**) SwinIR pretrained model, (**h**) SwinIR (train 1) and (**i**) SwinIR (train 2). Images from (**a**–**i**) correspond to the rows in Table 3 respectively.

**Figure 4:**
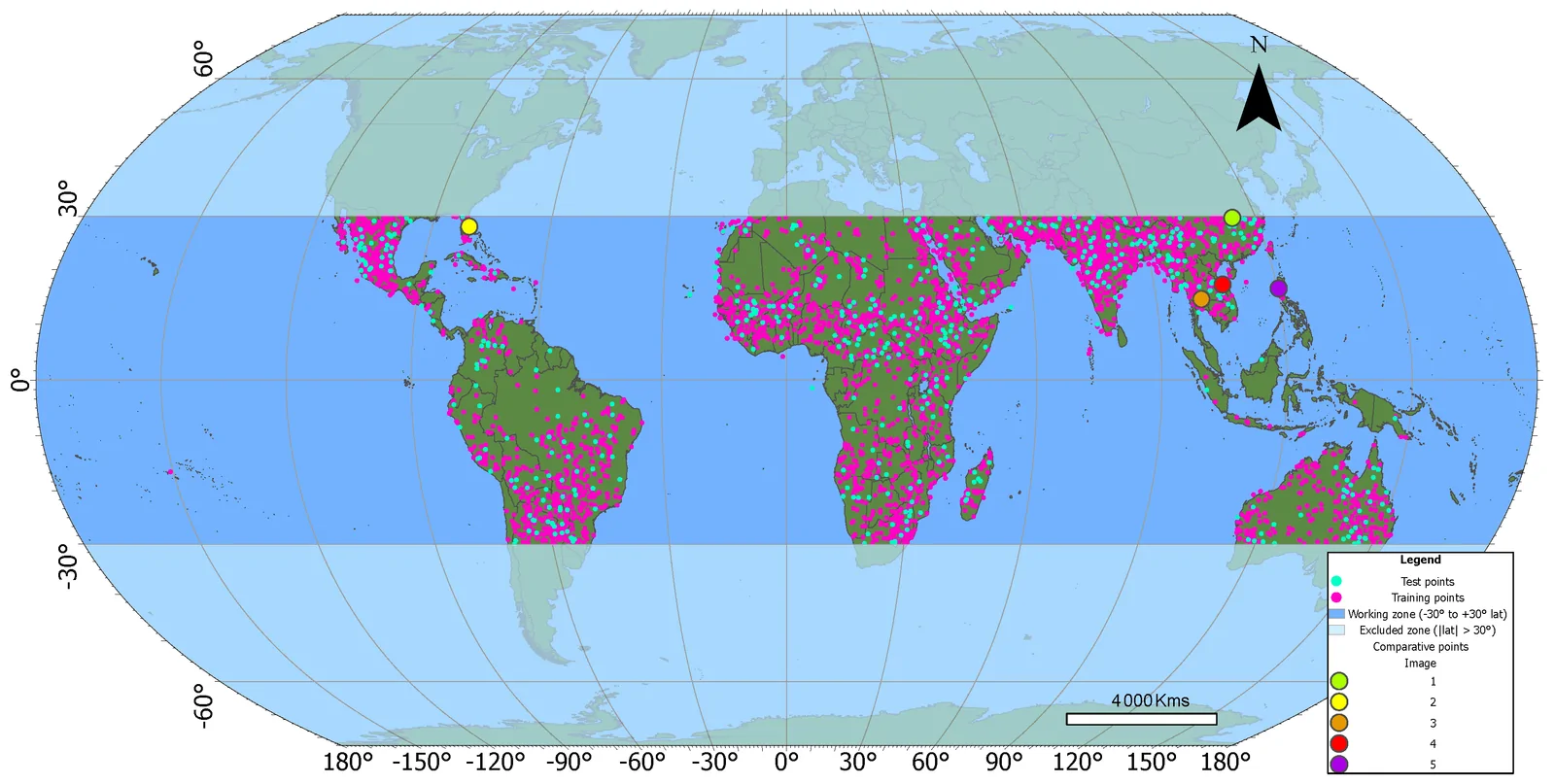
Geographic distribution of the working zones, excluded zones, training points, test points, and comparative points (1–5).

**Figure 5:**
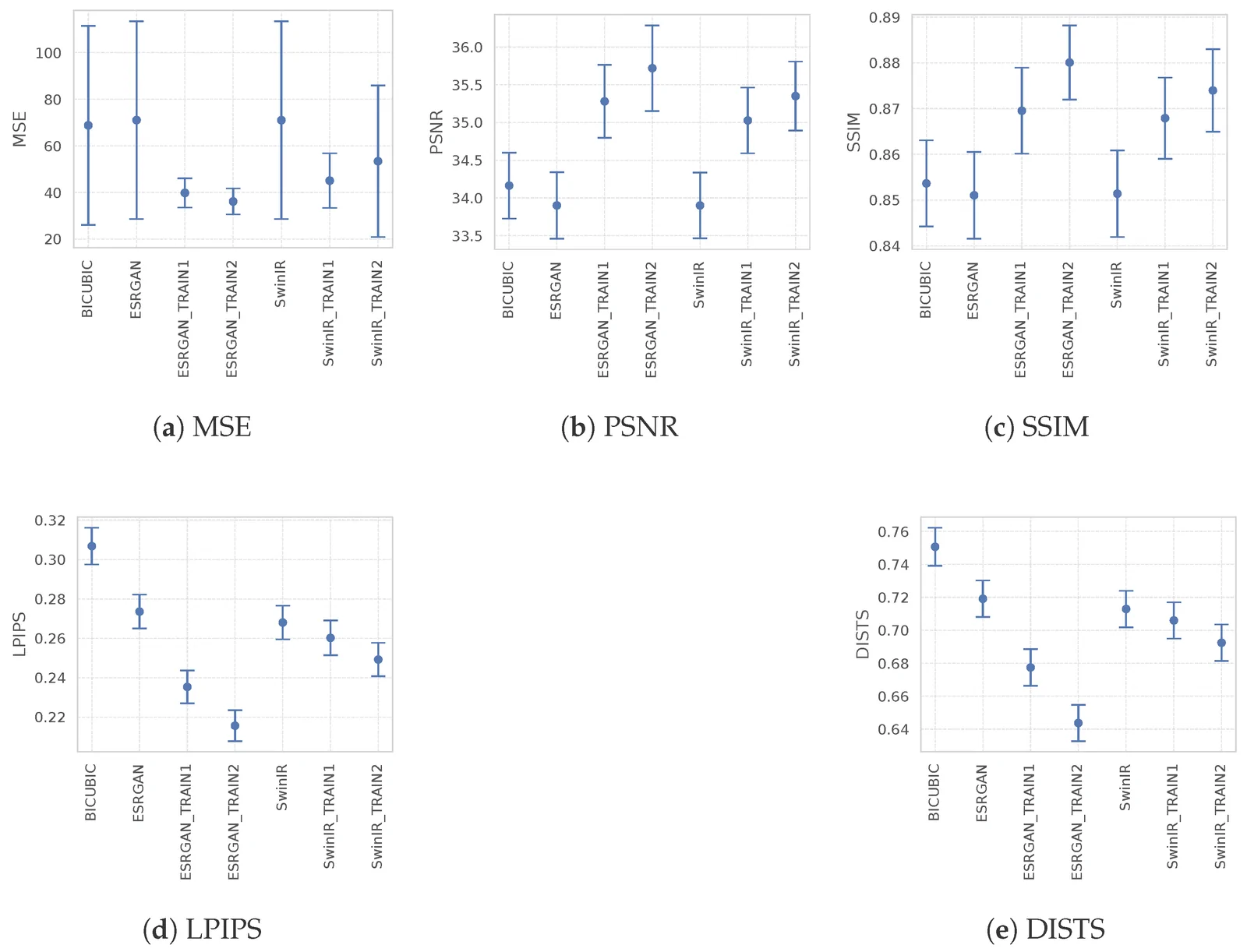
95% confidence intervals of performance metrics: (**a**) MSE, (**b**) PSNR, (**c**) SSIM, (**d**) LPIPS and (**e**) DISTS.

**Figure 6:**
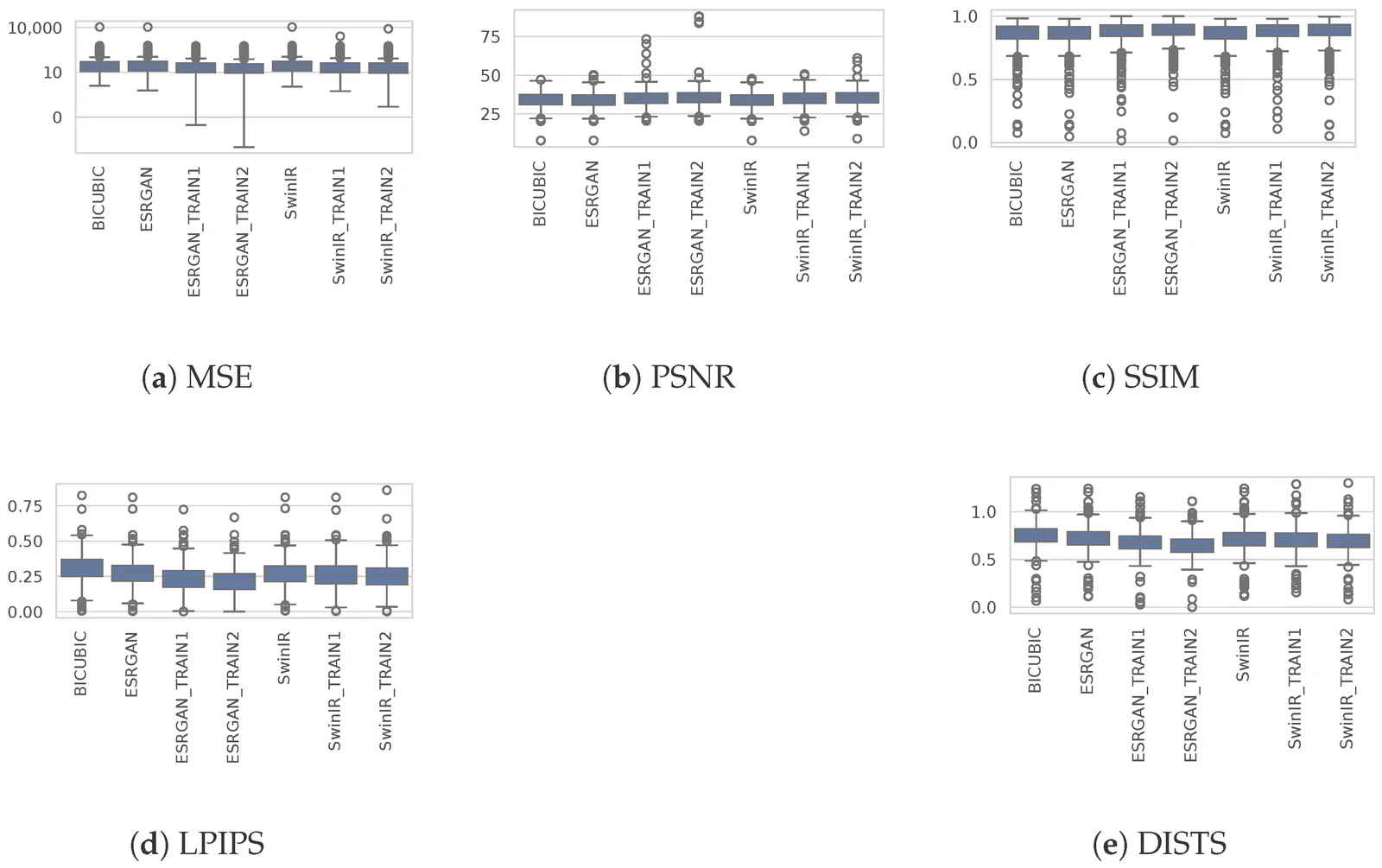
Boxplots of MSE, PSNR, SSIM, LPIPS, and DISTS values across the 500 independent test images for the evaluated models: (**a**) MSE, (**b**) PSNR, (**c**) SSIM, (**d**) LPIPS, and (**e**) DISTS.

**Figure 7:**
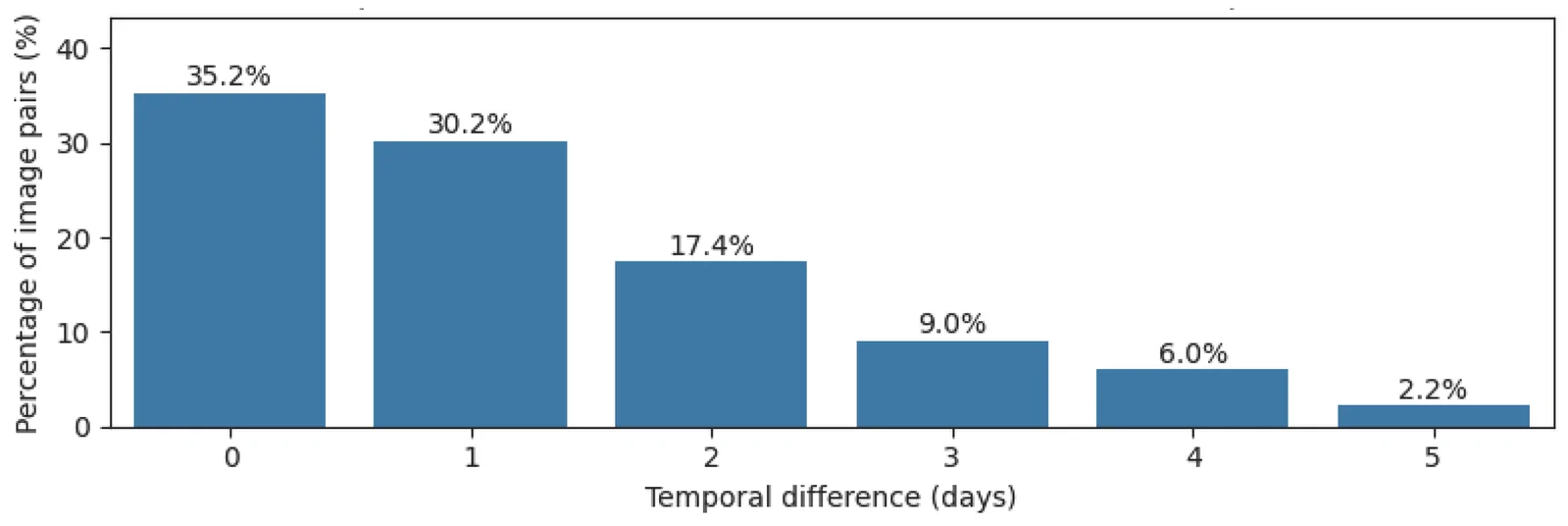
Temporal distribution of the acquisition-date differences in paired Landsat and Sentinel-2 images in the test dataset.

**Figure 8:**
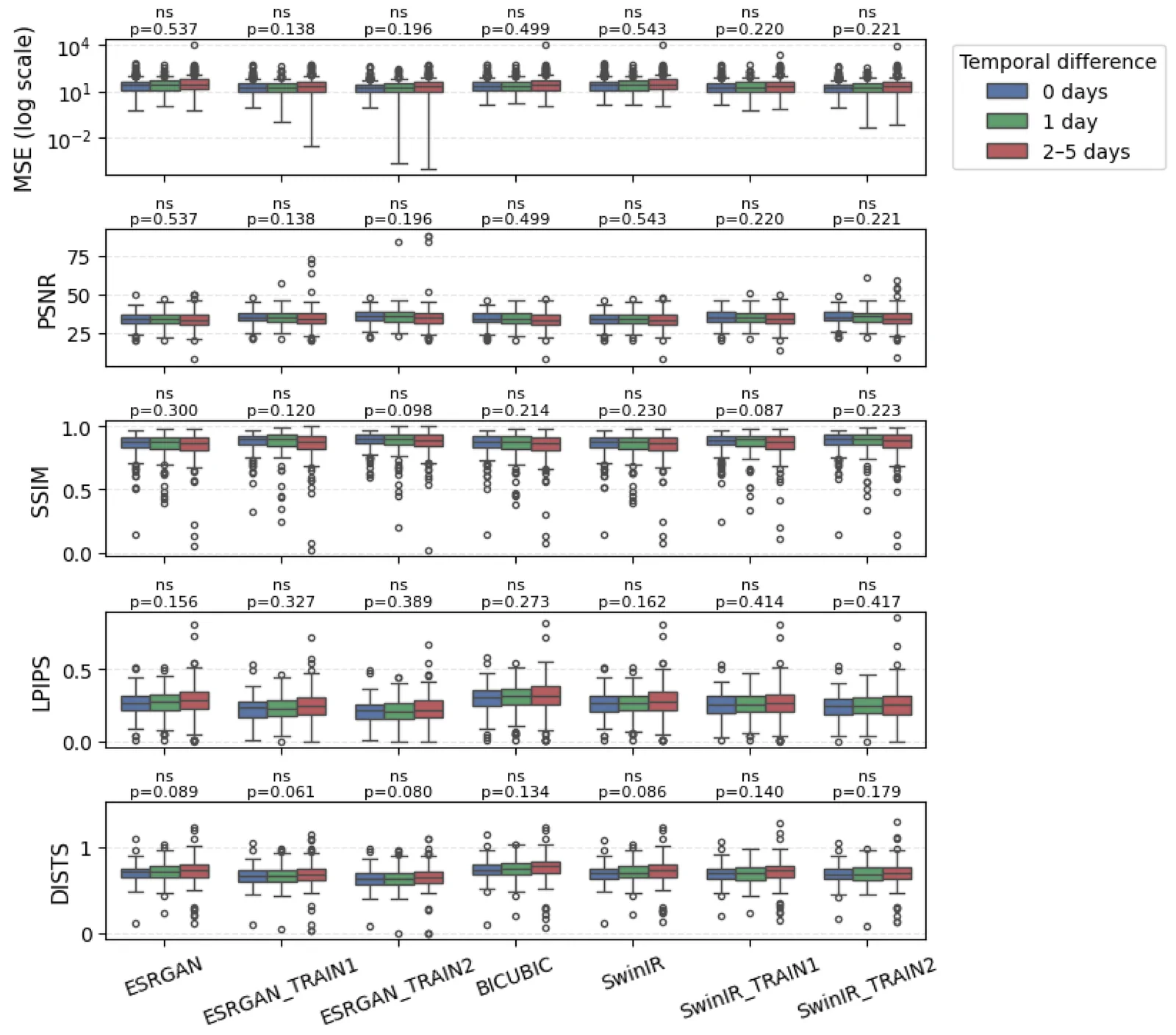
Distribution of MSE, PSNR, SSIM, LPIPS, and DISTS across the temporal-gap groups (0 days, 1 day, and 2–5 days) for the evaluated models. “ns” indicates a non-statistically significant difference (p>0.05) according to the Kruskal–Wallis test.

**Figure 9:**
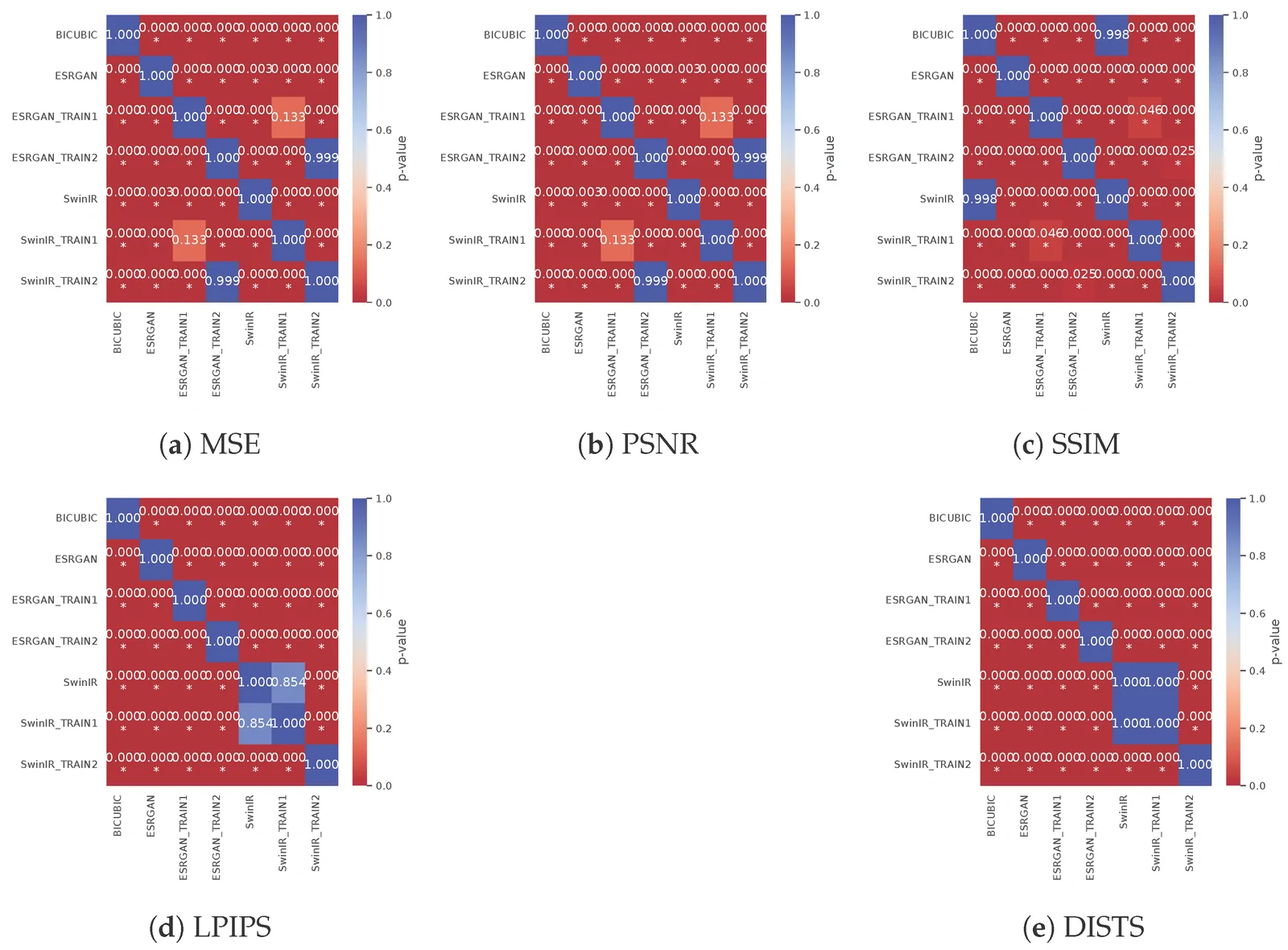
Pairwise *p*-value matrices from the post hoc Nemenyi test for (**a**) MSE, (**b**) PSNR, (**c**) SSIM, (**d**) LPIPS, and (**e**) DISTS. Lower *p*-values indicate statistically significant differences between model pairs (p<0.05), marked with an asterisk (*).

**Table 1:** Results of the D’Agostino–Pearson normality test (K^2^ statistic and *p*-value) for each evaluated metric and model. All distributions reject normality at α=0.05.

	Bicubic		ESRGAN		ESRGAN_T1		ESRGAN_T2	
Metric	K^2^	*p*-Value	K^2^	*p*-Value	K^2^	*p*-Value	K^2^	*p*-Value
MSE	1213.43	$3.22\times 10^{-264}$	1212.13	$6.17\times 10^{-264}$	511.29	$9.45\times 10^{-112}$	521.70	$5.18\times 10^{-114}$
PSNR	29.96	$3.12\times 10^{-7}$	25.19	$3.39\times 10^{-6}$	185.32	$5.74\times 10^{-41}$	441.52	$1.33\times 10^{-96}$
SSIM	368.89	$7.87\times 10^{-81}$	382.56	$8.47\times 10^{-84}$	412.52	$2.64\times 10^{-90}$	425.14	$4.81\times 10^{-93}$
LPIPS	26.64	$1.65\times 10^{-6}$	41.94	$7.82\times 10^{-10}$	32.37	$9.36\times 10^{-8}$	33.08	$6.57\times 10^{-8}$
DISTS	111.27	$6.88\times 10^{-25}$	76.01	$3.12\times 10^{-17}$	100.62	$1.41\times 10^{-22}$	114.58	$1.31\times 10^{-25}$
	**SwinIR**		**SwinIR_T1**		**SwinIR_T2**			
**Metric**	**K^2^**	***p*-value**	**K^2^**	***p*-value**	**K^2^**	***p*-value**		
MSE	+1212.00	$6.57\times 10^{-264}$	1019.18	$4.87\times 10^{-222}$	1209.79	$1.98\times 10^{-263}$		
PSNR	26.27	$1.97\times 10^{-6}$	16.14	$3.13\times 10^{-4}$	46.23	$9.16\times 10^{-11}$		
SSIM	377.68	$9.74\times 10^{-83}$	371.01	$2.74\times 10^{-81}$	420.59	$4.69\times 10^{-92}$		
LPIPS	49.03	$2.26\times 10^{-11}$	40.49	$1.61\times 10^{-9}$	65.98	$4.70\times 10^{-15}$		
DISTS	66.07	$4.50\times 10^{-15}$	45.39	$1.40\times 10^{-10}$	70.65	$4.55\times 10^{-16}$		

**Table 2:** Friedman test results per evaluation metric.

Metric	$\chi_{F}^{2}$	*p*-Value	Significant Differences
MSE	1258.42	<0.0001	Yes
PSNR	1258.42	<0.0001	Yes
SSIM	1496.26	<0.0001	Yes
LPIPS	2243.07	<0.0001	Yes
DISTS	2334.45	<0.0001	Yes

## Data Availability

The complete code and data used in this study are publicly available. The source code for the dataset generation pipeline and model training is hosted on GitHub at https://github.com/crialejo24/DOWNSCALING (accessed on 16 September 2026). The paired Landsat-Sentinel dataset (3000 training patches and 500 test patches) and the trained model weights for SwinIR and ESRGAN are available on Hugging Face at https://huggingface.co/datasets/Crialejo924/DOWNSCALING (accessed on 16 September 2026).

## Abbreviations

The following abbreviations are used in this manuscript:

CNN	Convolutional Neural Network
SWIN	Shifted WINdows transformer
SWINIR	SWIN transformer for Image Restoration
GAN	Generative Adversarial Network
ESRGAN	Enhanced Super-Resolution GAN
DIV2K	DIVerse 2K resolution dataset
RSTB	Residual Swin Transformer Block
PSNR	Peak Signal-to-Noise Ratio
MSE	Mean Squared Error
SSIM	Structural Similarity Index
LPIPS	Learned Perceptual Image Patch Similarity
DISTS	Deep Image Structure and Texture Similarity
RRDB	Residual-in-Residual Dense
ANOVA	ANalysis Of VAriance
GEE	Google Earth Engine
ESA	European Space Agency
NASA	National Aeronautics and Space Administration
